# Nowcasting for Real-Time COVID-19 Tracking in New York City: An Evaluation Using Reportable Disease Data From Early in the Pandemic

**DOI:** 10.2196/25538

**Published:** 2021-01-15

**Authors:** Sharon K Greene, Sarah F McGough, Gretchen M Culp, Laura E Graf, Marc Lipsitch, Nicolas A Menzies, Rebecca Kahn

**Affiliations:** 1 Bureau of Communicable Disease New York City Department of Health and Mental Hygiene Long Island City, NY United States; 2 Center for Communicable Disease Dynamics Department of Epidemiology Harvard TH Chan School of Public Health Boston, MA United States; 3 Genentech, Inc South San Francisco, CA United States; 4 Bureau of Epidemiology Services New York City Department of Health and Mental Hygiene Long Island City, NY United States; 5 Department of Global Health and Population Harvard TH Chan School of Public Health Boston, MA United States

**Keywords:** COVID-19, data quality, epidemiology, forecasting, infectious disease, morbidity and mortality trends, public health practice, surveillance

## Abstract

**Background:**

Nowcasting approaches enhance the utility of reportable disease data for trend monitoring by correcting for delays, but implementation details affect accuracy.

**Objective:**

To support real-time COVID-19 situational awareness, the New York City Department of Health and Mental Hygiene used nowcasting to account for testing and reporting delays. We conducted an evaluation to determine which implementation details would yield the most accurate estimated case counts.

**Methods:**

A time-correlated Bayesian approach called Nowcasting by Bayesian Smoothing (NobBS) was applied in real time to line lists of reportable disease surveillance data, accounting for the delay from diagnosis to reporting and the shape of the epidemic curve. We retrospectively evaluated nowcasting performance for confirmed case counts among residents diagnosed during the period from March to May 2020, a period when the median reporting delay was 2 days.

**Results:**

Nowcasts with a 2-week moving window and a negative binomial distribution had lower mean absolute error, lower relative root mean square error, and higher 95% prediction interval coverage than nowcasts conducted with a 3-week moving window or with a Poisson distribution. Nowcasts conducted toward the end of the week outperformed nowcasts performed earlier in the week, given fewer patients diagnosed on weekends and lack of day-of-week adjustments. When estimating case counts for weekdays only, metrics were similar across days when the nowcasts were conducted, with Mondays having the lowest mean absolute error of 183 cases in the context of an average daily weekday case count of 2914.

**Conclusions:**

Nowcasting using NobBS can effectively support COVID-19 trend monitoring. Accounting for overdispersion, shortening the moving window, and suppressing diagnoses on weekends—when fewer patients submitted specimens for testing—improved the accuracy of estimated case counts. Nowcasting ensured that recent decreases in observed case counts were not overinterpreted as true declines and supported officials in anticipating the magnitude and timing of hospitalizations and deaths and allocating resources geographically.

## Introduction

Timeliness is a key attribute of surveillance systems for reportable infectious diseases [1,2]. Timely surveillance data for COVID-19 are used by governments and communities to allocate resources and to decide when to tighten or loosen physical distancing and other prevention measures [3,4]. However, public health authorities track reportable diseases at a lag, given delays from infection to symptom onset, care seeking, specimen collection, laboratory testing, and reporting [5]. Monitoring prediagnostic data sources (eg, emergency department syndromic surveillance [6], internet searches and social media [7], participatory surveillance of self-reported symptoms [8], smart thermometers [9], etc) can improve timeliness at the expense of specificity, such as an inability to distinguish increases in respiratory illness attributable to influenza from COVID-19. Another approach that preserves specificity when monitoring COVID-19 disease trends is to leverage partially reported disease data, formally accounting for data lags.

The terms *nowcasting*, or predicting the present, and *hindcasting*, or predicting through the day prior to the present, describe a wide range of statistical adjustments used to fill in cases that are not yet reported, offering health officials a more up-to-date picture for situational awareness [10]. For example, researchers have assessed the potential to nowcast COVID-19 cases and deaths using Google Trends data available in near-real time [11], and have applied a range of modeling approaches that leverage reporting delays to estimate the number of not-yet-reported cases and deaths [12,13]. Using mathematical models to exploit COVID-19 transmission dynamics, nowcasting also has been extended to COVID-19 forecasting systems [14,15]. In a majority of these approaches, the nowcasting mechanism relies on accurately estimating the distribution of reporting delays; however, infectious disease transmission contains an important temporal component, in that incidence is correlated from one time point to the next, which has also been shown to improve nowcasting performance, including in COVID-19 applications [10,16].

We describe the use and evaluation of a time-correlated Bayesian nowcasting approach at the New York City (NYC) Department of Health and Mental Hygiene (DOHMH) during the first epidemic wave of COVID-19 to support real-time situational awareness and resource allocation. During the period from March to May 2020, approximately 203,000 laboratory-confirmed COVID-19 cases were reported to NYC DOHMH, peaking during the week of March 29, with approximately 5100 cases diagnosed per day [17]. Testing rates increased during this period as testing criteria at public health laboratories were relaxed, commercial and hospital laboratories developed testing capacity, and additional testing sites were opened and promoted [17].

## Methods

### Reportable Disease Surveillance Data

#### Persons Tested

Clinical and commercial laboratories are required to report all results, including positive, negative, and indeterminate results, for SARS-CoV-2 tests for New York State residents to the New York State Electronic Clinical Laboratory Reporting System (ECLRS) [18,19]. For NYC residents, ECLRS transmits reports to NYC DOHMH. These laboratory reports include specimen collection date and patient demographic information, including residential address.

For nowcasting persons newly tested, NYC DOHMH deduplicated laboratory reports, retaining the first report received (ie, report date) in ECLRS per person of a SARS-CoV-2 polymerase chain reaction (PCR) test. We retained the first specimen collection date for that associated test report date and the patient’s ZIP Code of residence at time of report.

ZIP Codes are collections of points constituting a mail delivery route. The United States Census Bureau developed ZIP Code Tabulation Areas (ZCTAs), which are aggregates of census blocks, to provide an areal representation of ZIP Codes. NYC DOHMH created a custom geography referred to as a modified ZCTA (modZCTA) by merging ZCTAs with populations of less than 3000 to an adjacent ZCTA with a larger population and merging interior ZCTAs with smaller populations to the surrounding ZCTA [20,21]. There are 177 modZCTAs within NYC.

#### Confirmed Cases

At NYC DOHMH, electronic laboratory reports are automatically standardized, and positive results indicating a confirmed case (ie, detection of SARS-CoV-2 RNA in a clinical specimen using a molecular amplification detection test) [22] are transmitted to the NYC DOHMH’s communicable disease surveillance database known as Maven (Conduent Public Health Solutions). For confirmed cases, the *diagnosis date* was defined as the specimen collection date of the first positive test. The *report date* was defined as the date the case was created in the disease surveillance database, which typically corresponded to the date the first positive test was reported to ECLRS.

Hospitalization status was ascertained by routinely matching patient identifiers for confirmed COVID-19 cases with hospitalized patients in supplemental data systems, including regional health information organizations, the New York State Hospital Emergency Response Data System, and NYC public hospitals [17]. For each hospitalized patient with a confirmed COVID-19 diagnosis, the hospital name for the most recent hospitalization in NYC was standardized to the name of a fully operational medical center. Patients with hospital discharge dates greater than 14 days prior to the collection date of their first positive PCR result were not considered hospitalized for COVID-19. The date of hospitalization ascertainment was not retained.

### Real-Time Nowcasting

NYC DOHMH nowcasted three outcomes (ie, confirmed cases, ever-hospitalized cases, and persons tested) among NYC residents at weekly increments; outcomes were nowcasted in real time through May 2020 on Mondays using reports received through the prior day on Sunday. Starting on March 24, 2020, nowcasts were conducted for all confirmed COVID-19 cases and restricted to the subset of confirmed COVID-19 cases among patients ever hospitalized. Starting on May 2, 2020, as testing became more widely available [23], nowcasts were conducted for persons newly tested by PCR for SARS-CoV-2. Each outcome was nowcasted citywide and also stratified by modZCTA of patient residence, to support targeting of community-based resources. Hospitalized cases were also nowcasted stratifying by health care facility, to support allocating resources to hospitals.

To account for reporting delays and the shape of the outcome-specific epidemic curve, we applied the R package Nowcasting by Bayesian Smoothing (NobBS), version 0.1.0 [10,24] (The R Foundation), to data for specimens collected or diagnoses during the 3 weeks prior to the nowcast through the date prior to the nowcast. Briefly, this approach corrects for underestimation of cases in real time caused by delays in reporting, learning the historical distribution of delays and relationship between cases in sequential time points to estimate the number of cases not yet reported. In performing stratified nowcasts, NobBS estimated the delay distribution citywide and the epidemic curve uniquely by stratum. Reports visualizing nowcast results were distributed weekly to DOHMH leadership for situational awareness.

We assumed an underlying Poisson distribution for case occurrence because this was the default setting in NobBS. The 3-week moving window was selected under the assumption that this length would adequately balance recency with stability. Although the optimal moving-window length was unknown in real time, given competing priorities during a pandemic, busy DOHMH officials would not have had adequate time to consider multiple nowcast versions with different window lengths as sensitivity analyses. The potential of the choice of moving-window length to considerably change nowcast estimates motivated a retrospective performance evaluation.

### Retrospective Nowcasting Evaluation

For the outcome of confirmed COVID-19 cases, we characterized the delay distribution between diagnosis and report, overall during the study period and by month of report, by median number of days, IQR, and 90^th^ percentile. We assessed the sensitivity of nowcasting results for patients diagnosed citywide during the period from March 22 to May 31, 2020—excluding cases diagnosed from March 1 to 21, given limited testing—to several choices: (1) day of week when the nowcast was performed, given outpatients with milder illness sought care and were diagnosed less frequently on weekends, when health care provider offices were typically closed or had more limited hours; (2) window length, given time-varying SARS-CoV-2 testing availability and uptake in NYC; and (3) assumed underlying distribution (ie, Poisson or negative binomial) for case occurrence. We generated Poisson regression models for the daily count by diagnosis date, separately for the entire study period and for every overlapping and nonoverlapping 2- and 3-week period, with and without weekends, used in the nowcasting evaluation. We checked the dispersion ratio for these Poisson regression models; dispersion ratios that were greater than 1 and statistically significant would indicate overdispersion and support instead using a negative binomial distribution. In addition, for nowcasting the number of cases stratified by modZCTA, we compared results using (1) the *strata* option in NobBS, which estimated the delay distribution citywide and epidemic curve separately for each modZCTA, versus estimating both the delay distribution and epidemic curve separately for each modZCTA and (2) 10,000 versus 3000 adaptations when optimizing the nowcasting algorithm [10].

Data for the evaluation were frozen as of June 30, 2020, capturing reports received through 1 month after the end of the assessment period. We mimicked prospective surveillance at weekly intervals and daily temporal resolution, retaining the number of estimated cases for each of the prior 7 days (ie, 1-7-day hindcasts). We used the mean absolute error and the average daily relative root mean square error across all days evaluated to compare the point estimate of the number of daily hindcasted cases over the time series with the true number of cases reported. For each of these metrics, lower numbers indicate better performance of the hindcast. We also assessed the 95% prediction interval coverage (ie, the proportion of days during the study period when the 95% prediction interval included the true number of cases) [10], which should ideally be 95%.

This work was reviewed and deemed as public health surveillance that is nonresearch by the DOHMH Institutional Review Board. Line-level data, as required for nowcasting using NobBS, are not publicly available in accordance with patient confidentiality and privacy laws.

## Results

**Figure 1:**
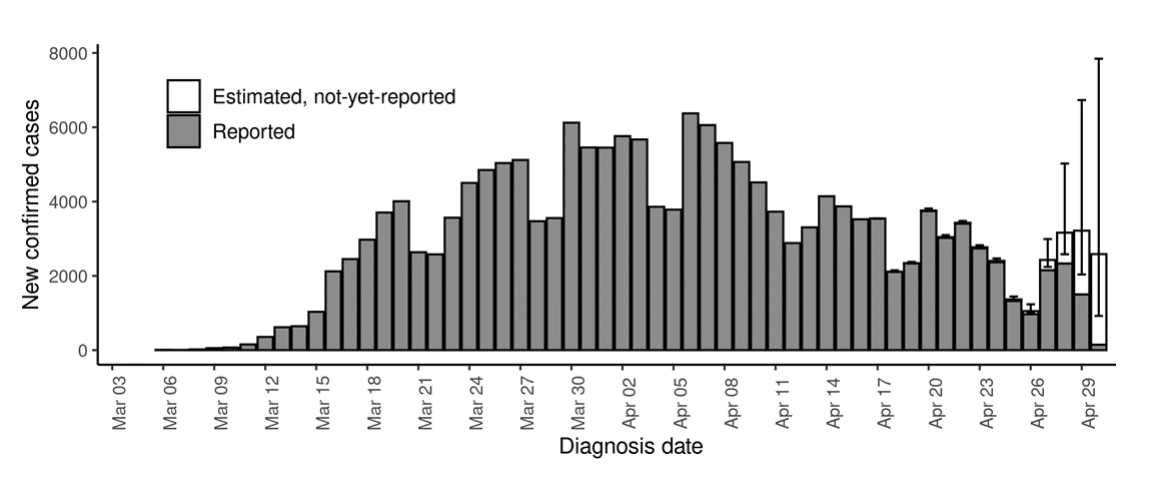
Example hindcast visualization of epidemic curve of reported and estimated but not-yet-reported number of confirmed cases among New York City residents diagnosed with COVID-19, from March 1 to April 30, 2020. Illustrative hindcast performed using cases reported through April 30, 2020 (ie, a Thursday), a 2-week moving window, and a negative binomial distribution.

Among confirmed COVID-19 cases residing in NYC and diagnosed during the period from March to May 2020, the median delay between specimen collection and report was 2 days (IQR 1-4; 90^th^ percentile 7). By month of report for diagnoses during the period of March to May 2020, the median number of days for this delay for reports received in March 2020 was 2 (IQR 1-4; 90^th^ percentile 7), in April was also 2 (IQR 1-4; 90^th^ percentile 7), in May was 2 (IQR 1-3; 90^th^ percentile 5), and in June, given the study period included cases diagnosed through May, extended to 7 (IQR 4-19; 90^th^ percentile 62). Hindcasts were performed weekly on Mondays in real time, with results visualized for DOHMH leadership (eg, see Figure 1).

**Figure 2:**
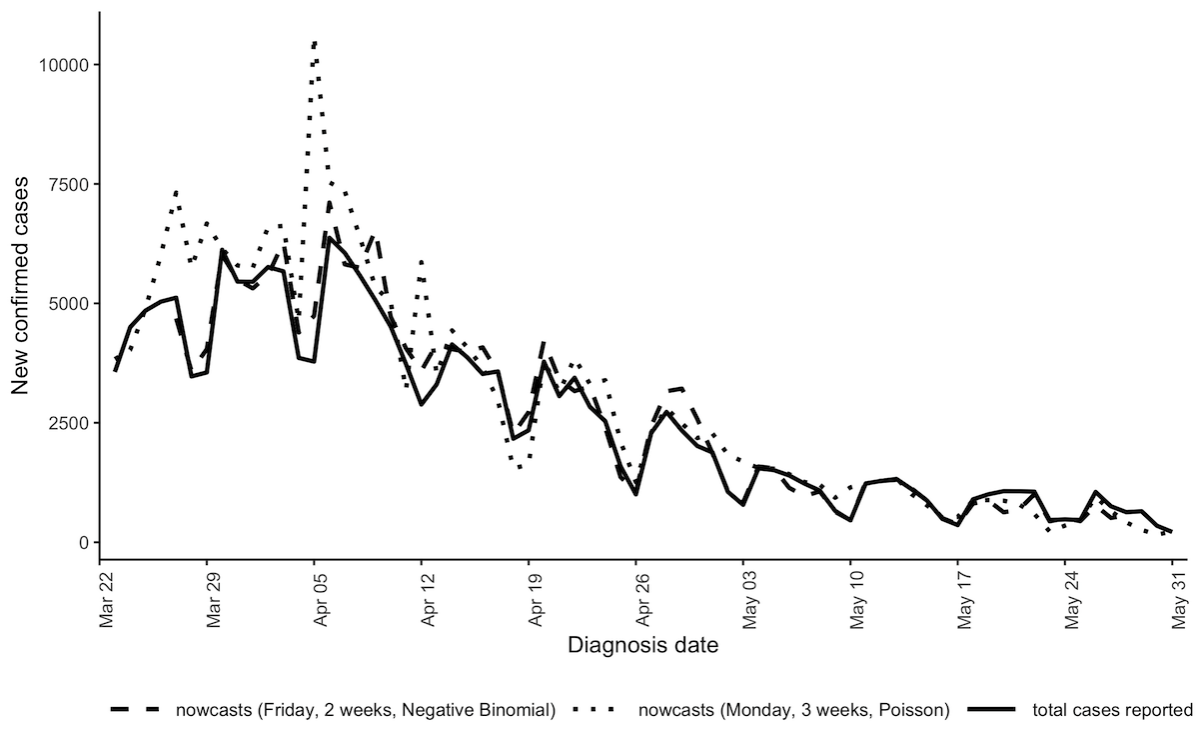
Comparison of 7-day hindcasts conducted on Fridays with a 2-week window and negative binomial distribution, and 7-day hindcasts conducted on Mondays with a 3-week window and Poisson distribution. Total cases reported as of June 30, 2020, are shown with a black line.

However, the retrospective performance evaluation determined that real-time hindcasts on Mondays using a 3-week window and an assumed Poisson distribution more often overestimated than underestimated the number of not-yet-reported cases and resulted in overly narrow 95% prediction intervals (see Figure 2 and Figure S1 in Multimedia Appendix 1). Subsequent results focus on two scenarios: the scenario that was used in real time (ie, a 3-week moving window and Poisson distribution) and the scenario that would have performed best had it been used in real time (ie, a 2-week moving window and negative binomial distribution).

We found that citywide hindcasts with a 2-week moving window and a negative binomial distribution had a 44% lower mean absolute error, a 31% lower relative root mean square error, and 0.65 higher 95% prediction interval coverage than hindcasts conducted with a 3-week moving window or with a Poisson distribution (see Table 1 as well as Table S1 and Figures S1 and S2 in Multimedia Appendix 1). Poisson regression models for daily count data for the entire study period and for each 2- and 3-week period evaluated were overdispersed (median dispersion ratio 97.5, all *P*<.05), which explains the better performance of the negative binomial distribution. While dispersion ratios were lower for analyses restricted to weekdays (median ratio of 32.5 vs 150 for all days), all were greater than 1, indicating overdispersion.

**Table 1:** Performance measures for hindcasting approaches applied to citywide case counts of New York City residents diagnosed with COVID-19, from March 22 to May 31, 2020.

Approach and sensitivity analyses		All days			Weekdays only		
		Mean absolute error	Relative root mean square error	95% prediction interval coverage	Mean absolute error	Relative root mean square error	95% prediction interval coverage
**Base scenario used in near-real time by NYC DOHMH^a^, using 3-week window with Poisson distribution**							
	All days	544	0.20	0.16	559	0.19	0.16
	Hindcasting each Monday for the previous Monday-Sunday	556	0.25	0.14	338	0.12	0.20
**Day-of-week hindcasting was performed for previous 7-day period, using 2-week window with negative binomial distribution**							
	All days	306	0.14	0.81	258	0.10	0.84
	Monday	336	0.20	0.86	183	0.07	0.82
	Tuesday	335	0.16	0.83	233	0.08	0.84
	Wednesday	307	0.14	0.81	275	0.11	0.87
	Thursday	271	0.11	0.81	257	0.11	0.84
	Friday	255	0.10	0.75	267	0.11	0.84
	Saturday	260	0.11	0.73	267	0.11	0.80
	Sunday	372	0.16	0.87	273	0.10	0.88

^a^NYC DOHMH: New York City Department of Health and Mental Hygiene.

Hindcasts conducted toward the end of the week (ie, Thursday to Saturday) performed better than hindcasts performed earlier in the week, presumably as they had the furthest distance from the weekends. Weekends had lower overall case counts than weekdays (see Figure 1). Until mid-May, hindcasts more often overestimated than underestimated true case counts, whereas at the end of May hindcasts more often underestimated case counts, reflecting changes in the delay distribution over time (see Figure 2 and Figure S3 in Multimedia Appendix 1).

To minimize day-of-week effects that were most prominent on weekends, we also restricted performance analysis to hindcasts of cases on weekdays only, which resulted in better metrics, as expected (see Table 1 and Table S1 in Multimedia Appendix 1). The hindcasts restricted to estimating case counts for weekdays with a 2-week moving window and negative binomial distribution also performed better than the hindcasts with a 3-week moving window and Poisson distribution, with 54% lower mean absolute error, 46% lower relative root mean square error, and 0.69 higher 95% prediction interval coverage (see Table 1 and Table S1 in Multimedia Appendix 1). Performance metrics were similar across days the hindcasts were conducted, with Mondays having the lowest mean average error and relative root mean square error, as expected given the 2 additional days between the last day reported (ie, Friday) and the day the hindcast was conducted (ie, Monday). On weekdays during the study period, the average daily case count after data lags resolved was 2914, the average hindcasted case count with a 2-week window and negative binomial distribution conducted on Mondays was 2878, and the mean absolute error was 183. A combination of the window length and underlying distribution influenced the performance of the mean absolute error and relative root mean square error metrics, with larger differences occurring between different windows with the same distribution than between different distributions with the same window. On the other hand, the distribution was the primary driver for differences in the 95% prediction interval coverage (ie, differences were larger between analyses with different distributions than between analyses with the same distribution and different windows).

For hindcasts at the modZCTA level, a 2-week moving window and negative binomial distribution performed best across all metrics evaluated (see Table 2 and Table S1 in Multimedia Appendix 1), although the prediction interval coverage for the nowcasts with a Poisson distribution was higher than for citywide hindcasts. The hindcasts that assumed a citywide delay distribution performed slightly better than hindcasts that assumed different distributions by modZCTA. Metrics for 3000 versus 10,000 adaptations were essentially the same.

**Table 2:** Performance measures for hindcasting approaches in Nowcasting by Bayesian Smoothing (NobBS), applied to case counts of New York City residents diagnosed with COVID-19 from March 22 to May 31, 2020, stratified by modified ZIP Code Tabulation Area (modZCTA) of residence.

Approach and sensitivity analyses		All days							Weekdays only				
		Mean absolute error		Relative root mean square error		95% prediction interval coverage		Mean absolute error		Relative root mean square error		95% prediction interval coverage	
**Base scenario used in near-real time by NYC DOHMH^a,b^**													
	3-week Poisson (10,000 adaptations)		3.82		0.37		0.84		2.75		0.18		0.84
	3-week Poisson (3000 adaptations)		3.83		0.37		0.84		2.76		0.18		0.84
	2-week negative binomial (10,000 adaptations)		2.92		0.33		0.93		2.09		0.15		0.93
	2-week negative binomial (3000 adaptations)		2.93		0.34		0.93		2.08		0.15		0.93
**Conducting hindcasts on Fridays^c^**													
	2-week negative binomial		2.62		0.22		0.94		2.98		0.25		0.95
**Estimate delay distribution separately by modZCTA^d^**													
	2-week negative binomial		3.55		0.36		0.94		2.57		0.21		0.95

^a^NYC DOHMH: New York City Department of Health and Mental Hygiene.

^b^The approach used the *strata* option in NobBS, which estimated the delay distribution citywide and epidemic curve separately for each modZCTA, conducted on Mondays

^c^The approach used the *strata* option in NobBS, which estimated the delay distribution citywide and epidemic curve separately for each modZCTA, conducted on Fridays.

^d^The approach involved estimating both the delay distribution and epidemic curve separately for each modZCTA conducted on Mondays.

## Discussion

### Principal Findings

NYC DOHMH improved situational awareness of COVID-19 testing and cases during the first epidemic wave in near-real time by applying NobBS, a readily accessible nowcasting and hindcasting method. As a result of the retrospective performance evaluation, to improve nowcast accuracy prospectively effective August 2020, we implemented the following changes to the nowcasting approach: (1) we used a negative binomial case distribution instead of a Poisson; (2) we linked the determination of the moving-window length (ie, 2 or 3 weeks) to the 90^th^ percentile of the lag between specimen collection and report for reports received in the most recent week, choosing 3 weeks if the 90^th^ percentile of the lag distribution is more than 14 days; and (3) we suppressed nowcasting results for specimens collected on weekends, given lack of adjustment for day-of-week effects. The evaluation supported the results of nowcasting conducted on any weekday.

Despite a mature electronic laboratory reporting system and strong informatics infrastructure and data cleaning procedures at NYC DOHMH, input data available for nowcasting had several limitations. First, for records with long lags between specimen collection and report, as long as the specimen was reported to have been collected during the pandemic period, it was not possible to distinguish long lags attributable to true delays in testing or reporting—and, thus, informative to the delay distribution—from long lags attributable to laboratory data entry errors in specimen collection dates. Second, nowcasting by patient modZCTA of residence relied on accurate laboratory reporting of patient address. For example, 1 week of real-time nowcasting results were biased when, for a batch of reports, one commercial laboratory misreported its own address as the residential address of all patients tested. Third, patient hospitalization status was largely ascertained by matching administrative records. To allow time for record matching, hospitalization nowcasts were conducted at a 3-day lag, limiting the real-time availability of results. Furthermore, records from certain facilities were unavailable in near-real time, so nowcasts of hospitalizations by patient residence and by facility were subject to spatial bias, although still considered by DOHMH leadership to be useful for situational awareness.

This version of NobBS (ie, version 0.1.0) also had several limitations when applied for nowcasting COVID-19 in NYC. First, there was no built-in functionality in NobBS to account for observable factors influencing data lags, including day-of-week and holiday effects in outpatient testing, and time-varying testing backlogs at specific laboratories differentially processing specimens for residents across neighborhoods. A recent COVID-19 nowcasting study in Bavaria, which adapted certain modeling elements from NobBS, found that modeling a weekday effect improved nowcast performance [16]. Given the substantial differences in diagnoses on weekdays compared with weekends, similar adjustments would likely benefit NYC nowcasts but were unavailable in NobBS. Similarly, there was no functionality to account for temporal trends in testing (eg, the time-varying ratio of number of tests performed to number of cases detected). Third, while 95% prediction intervals reflected uncertainty in the nowcasts themselves—encompassing uncertainty in the estimation of the delay distribution as well as in the time evolution of the epidemic curve—they did not reflect uncertainty introduced by the user-specified window length. Fourth, in generating geographically stratified nowcasts, the *strata* option in NobBS estimated the delay distribution citywide and epidemic curve separately for each modZCTA or health care facility stratum. For a highly transmissible infectious disease, nowcasting performance might be improved by considering spatial relationships across geographic strata, including spatial autocorrelation. Finally, although government officials have demonstrated interest in publicizing test percent positivity by report date [25,26], which can be biased by data lags, NobBS did not have functionality to nowcast percentages as an outcome. NobBS could be used to separately nowcast persons testing positive and negative and then to calculate test percent positivity, but there is no functionality to appropriately account for the separate uncertainties in the numerator and denominator of this percentage.

### Practice Implications

When tracking ongoing outbreaks using epidemic curves, public health officials recognize that data for recent days are incomplete because of reporting delays. Data lags can make it difficult for policy makers to discern in near-real time whether apparent decreases in recent case counts are the result of public health interventions, such as social distancing guidelines.

NYC DOHMH filled in COVID-19 epidemic curves using NobBS, which helped ensure that recent decreases in observed case counts were not overinterpreted as true declines in disease and supported the continuation of policies to reduce transmission. Nowcasted citywide case counts supported situational awareness and assisted DOHMH leadership in anticipating the magnitude and timing of hospitalizations and deaths. Nowcasting hospitalizations by health care facility was useful in helping to route patient transports and avoid overburdening facilities.

As the COVID-19 pandemic continues, state and local health departments should incorporate nowcasting into their workflows. This performance evaluation led to analytic improvements in place for the second wave of COVID-19 in NYC, including the use of a more suitable underlying distribution for case occurrence, a dynamic window length to account for periods with an extended lag distribution, and suppression of diagnoses on weekends to avoid biased trend estimates. Nowcasted case counts can also be used as inputs for near-real time estimates of other outbreak monitoring metrics, including the time-varying reproduction number [27] and doubling times [28]. Further evaluations are warranted to assess nowcasting performance during different COVID-19 epidemic phases and across jurisdictions experiencing a variety of data lag distributions, including more extensive reporting delays [29], and for additional outcomes, such as deaths.

## Appendix

Multimedia Appendix 1 Supplemental table and figures.

## Abbreviations

DOHMH: Department of Health and Mental Hygiene
ECLRS: Electronic Clinical Laboratory Reporting System
modZCTA: modified ZIP Code Tabulation Area
NobBS: Nowcasting by Bayesian Smoothing
NYC: New York City
PCR: polymerase chain reaction
ZCTA: ZIP Code Tabulation Area
