# Supplementary material for: Nowcasting for Real-Time COVID-19 Tracking in New York City: An Evaluation Using Reportable Disease Data From Early in the Pandemic
Source: JMIR Public Health Surveill. 2021 Jan 15;7(1):e25538. doi: 10.2196/25538 (PMC7812916; doi:10.2196/25538)

**Multimedia Appendix 1 (Supplemental Table and Figures).**

**Nowcasting for Real-Time COVID-19 Tracking in New York City:**

**An Evaluation Using Reportable Disease Data From Early in the Pandemic**

doi: 10.2196/25538

**Table S1**. Performance measures for additional hindcasting approaches applied to citywide case counts of New York City residents diagnosed with COVID-19, March 22–May 31, 2020.

The first set of values represents the metric across all days hindcasted, and the second assesses only weekdays. For each method and metric, the best performing day of week is in red.

|  | | All days | | | Weekdays only | | |
| --- | --- | --- | --- | --- | --- | --- | --- |
| Method | Day of week hindcast conducted | Mean absolute error | Relative root mean square error | 95% prediction interval coverage | Mean absolute error | Relative root mean square error | 95% prediction interval coverage |
| 1-week window, negative binomial | **All** | **359** | **0.14** | **0.54** | **390** | **0.12** | **0.51** |
|  | Monday | 405 | 0.20 | 0.39 | 358 | 0.12 | 0.24 |
|  | Tuesday | 355 | 0.15 | 0.51 | 369 | 0.11 | 0.42 |
|  | Wednesday | 375 | 0.13 | 0.62 | 440 | 0.14 | 0.53 |
|  | Thursday | 338 | 0.11 | 0.65 | 399 | 0.12 | 0.62 |
|  | Friday | 343 | 0.12 | 0.60 | 419 | 0.14 | 0.62 |
|  | Saturday | 267 | 0.10 | 0.62 | 314 | 0.11 | 0.76 |
|  | Sunday | 415 | 0.16 | 0.41 | 432 | 0.13 | 0.40 |
| 2-week window, negative binomial | **All** | **306** | **0.14** | **0.81** | **258** | **0.10** | **0.84** |
|  | Monday | 336 | 0.20 | 0.86 | 183 | 0.07 | 0.82 |
|  | Tuesday | 335 | 0.16 | 0.83 | 233 | 0.08 | 0.84 |
|  | Wednesday | 307 | 0.14 | 0.81 | 275 | 0.11 | 0.87 |
|  | Thursday | 271 | 0.11 | 0.81 | 257 | 0.11 | 0.84 |
|  | Friday | 255 | 0.10 | 0.75 | 267 | 0.11 | 0.84 |
|  | Saturday | 260 | 0.11 | 0.73 | 267 | 0.11 | 0.80 |
|  | Sunday | 372 | 0.16 | 0.87 | 273 | 0.10 | 0.88 |
| 2-week window, Poisson | **All** | **367** | **0.15** | **0.24** | **372** | **0.14** | **0.26** |
|  | Monday | 380 | 0.20 | 0.29 | 193 | 0.08 | 0.34 |
|  | Tuesday | 444 | 0.18 | 0.25 | 417 | 0.14 | 0.33 |
|  | Wednesday | 393 | 0.16 | 0.24 | 421 | 0.16 | 0.22 |
|  | Thursday | 374 | 0.14 | 0.16 | 457 | 0.17 | 0.18 |
|  | Friday | 307 | 0.13 | 0.22 | 371 | 0.15 | 0.20 |
|  | Saturday | 358 | 0.14 | 0.24 | 468 | 0.18 | 0.18 |
|  | Sunday | 320 | 0.13 | 0.30 | 302 | 0.11 | 0.32 |
| 3-week window, negative binomial | **All** | **608** | **0.21** | **0.78** | **542** | **0.16** | **0.82** |
|  | Monday | 627 | 0.27 | 0.79 | 358 | 0.12 | 0.72 |
|  | Tuesday | 653 | 0.23 | 0.83 | 477 | 0.13 | 0.84 |
|  | Wednesday | 609 | 0.21 | 0.79 | 572 | 0.17 | 0.87 |
|  | Thursday | 532 | 0.17 | 0.78 | 536 | 0.16 | 0.87 |
|  | Friday | 534 | 0.17 | 0.73 | 570 | 0.18 | 0.84 |
|  | Saturday | 579 | 0.18 | 0.73 | 690 | 0.21 | 0.78 |
|  | Sunday | 706 | 0.24 | 0.80 | 602 | 0.17 | 0.80 |
| 3-week window, Poisson | **All** | **544** | **0.20** | **0.16** | **559** | **0.19** | **0.16** |
|  | Monday | 556 | 0.25 | 0.14 | 338 | 0.12 | 0.20 |
|  | Tuesday | 601 | 0.22 | 0.16 | 579 | 0.17 | 0.16 |
|  | Wednesday | 538 | 0.20 | 0.14 | 590 | 0.20 | 0.16 |
|  | Thursday | 519 | 0.18 | 0.14 | 614 | 0.21 | 0.16 |
|  | Friday | 477 | 0.17 | 0.19 | 545 | 0.20 | 0.18 |
|  | Saturday | 591 | 0.20 | 0.21 | 748 | 0.26 | 0.13 |
|  | Sunday | 525 | 0.19 | 0.16 | 524 | 0.17 | 0.12 |

**Figure S1**. Estimates (dotted black line) with 95% confidence bounds (grey) for hindcasts conducted on Mondays, with a 3-week window and Poisson distribution, and total cases reported (solid black line).


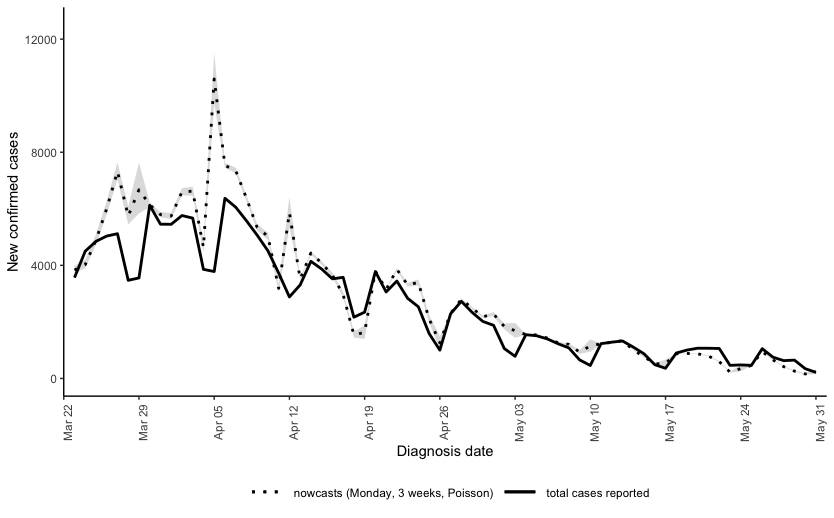


**Figure S2**. Estimates (dashed black line) with 95% confidence bounds (grey) for hindcasts conducted on Fridays, with a 2-week window and negative binomial distribution, and total cases reported (solid black line).


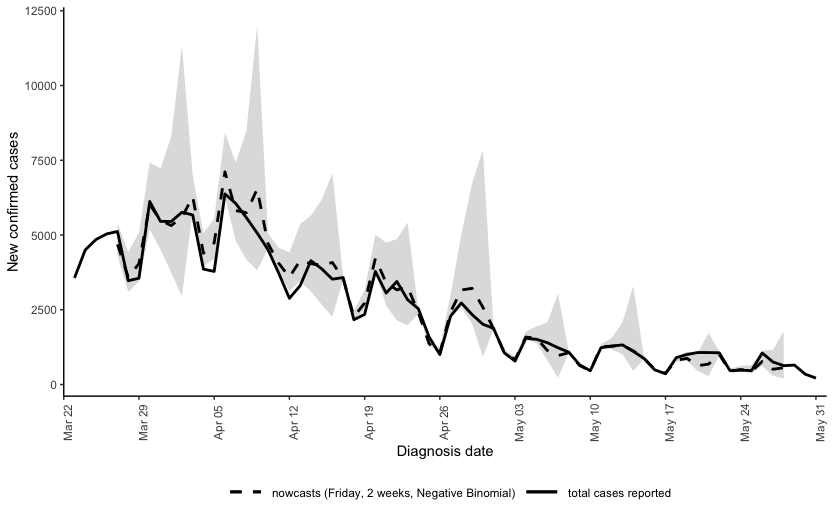


**Figure S3**. Median (interquartile range) of delays by week


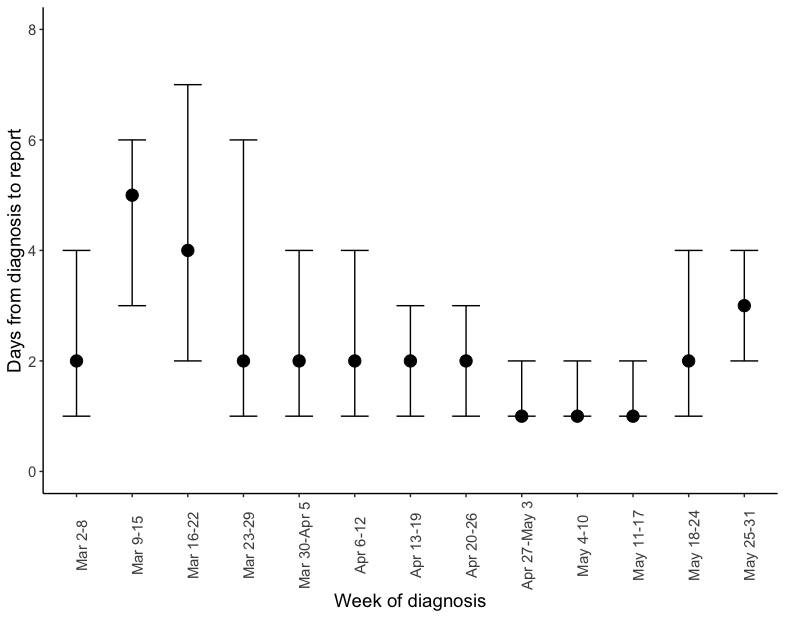

Supplement: Multimedia Appendix 1 [file publichealth_v7i1e25538_app1.docx]
